# DNA Damage Stress: Cui Prodest?

**DOI:** 10.3390/ijms20051073

**Published:** 2019-03-01

**Authors:** Nagendra Verma, Matteo Franchitto, Azzurra Zonfrilli, Samantha Cialfi, Rocco Palermo, Claudio Talora

**Affiliations:** Department of Molecular Medicine, Sapienza University of Rome, 00161 Rome, Italy; nagendra.verma@uniroma1.it (N.V.); matteo.franchitto@uniroma1.it (M.F.); azzurra.zonfrilli@uniroma1.it (A.Z.); samantha.cialfi@uniroma1.it (S.C.); rocco.palermo@uniroma1.it (R.P.)

**Keywords:** cell cycle checkpoints, genomic instability, G2-arrest, cell death, repair of DNA damage, adaptation

## Abstract

DNA is an entity shielded by mechanisms that maintain genomic stability and are essential for living cells; however, DNA is constantly subject to assaults from the environment throughout the cellular life span, making the genome susceptible to mutation and irreparable damage. Cells are prepared to mend such events through cell death as an extrema ratio to solve those threats from a multicellular perspective. However, in cells under various stress conditions, checkpoint mechanisms are activated to allow cells to have enough time to repair the damaged DNA. In yeast, entry into the cell cycle when damage is not completely repaired represents an adaptive mechanism to cope with stressful conditions. In multicellular organisms, entry into cell cycle with damaged DNA is strictly forbidden. However, in cancer development, individual cells undergo checkpoint adaptation, in which most cells die, but some survive acquiring advantageous mutations and selfishly evolve a conflictual behavior. In this review, we focus on how, in cancer development, cells rely on checkpoint adaptation to escape DNA stress and ultimately to cell death.

## 1. Introduction

While questionable, one of the most well-known and widely reported aspect in cancer biology is the acquisition of genetic mutations that underlie cell transformation and tumor progression. From this perspective, cell transformation is a genetic process of tumor cells adapted to stressful environmental conditions; if to ‘cell adaptation’ can be conferred the Darwinian concept to respond to life’s needs for survival, the nature of what adaptation means for tumor cells is extremely elusive. Either physical or chemical environmental agents can cause DNA damage and consequently genetic mutations that promote cell transformation.

Examples of physical agents promoting mutations are ionizing radiation, ultraviolet light present in sunlight which can promote the estimated rate of up to 10,000 DNA lesions per cell per day [1,2]; chemical agents such as benzo(a)pyrene B(a)P, 7,12-dimethylbenz[a]anthracene (DMBA), that generate DNA adducts, leading to mutations [3]. Beside exogenously, DNA damage can also occur endogenously as cells divide, with tens of thousands events every day in each single cell [2]. Thus, DNA damage might potentially affect the function of central regulators of many biological processes, ultimately leading to cancer development. Additionally, infectious pathogens elicit an oncogenic spiral that is one of the causes of cancer development [4]. If we assess the concept that ‘adaptation’ means the optimization of the phenotype whereby the organism acquires changes that increase its survival and reproductive success, when this concept is applied to cell transformation it remains extremely vague. Although this concept is suitable for viral carcinogenesis that hijacking cellular pathways promotes the survival and proliferation of infected cells, in a multicellular organism, cells do not need to adapt their phenotype to a non-permissive environment. Unquestionably, in multicellular organisms, cells are immersed in growth conditions favorable to their replication. However, there is an obvious difference in the relationship between adaptation and environment in unicellular versus multicellular organisms. Life and replication in unicellular organisms are dependent on the conditions present in the environment and they survive if they are able to adapt to environmental changes. In sharp contrast, in multicellular organisms cell division is tightly regulated to control cell shape, tissue patterns, and morphogenesis [5], although cells are typically immersed in permissive environmental conditions. Preservation of the integrity of multicellular organisms relies on these extra layers of developmental control that function to restrain cellular proliferation that may change in response to environmental or intracellular stress signals. This implies that, as previously defined [6,7], cancer cells arise from cells adapted to respond to holistic control system and the escape from these host defense mechanisms represents an important strategy for cell transformation.

## 2. Cell Cycle Surveillance System

**Figure 1:**
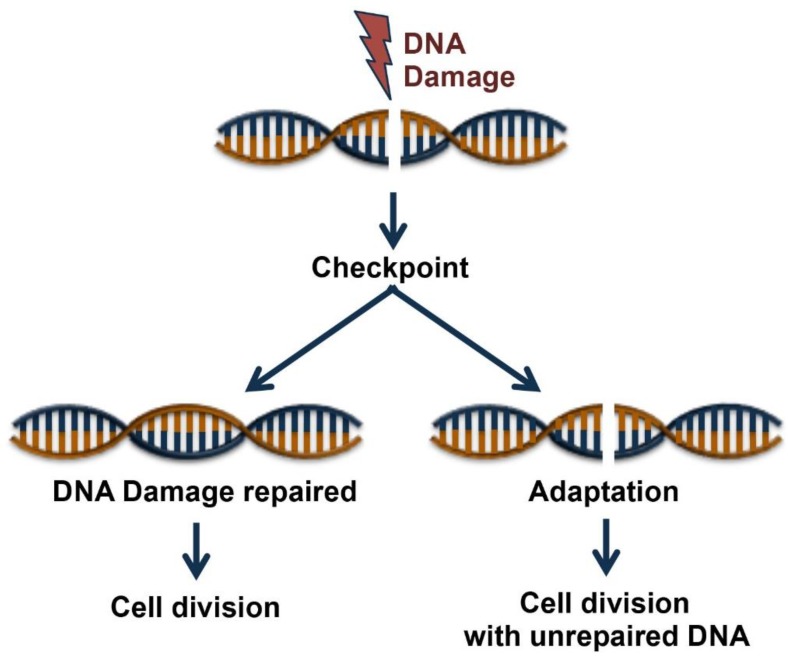
Cell fates following DNA Damage. Cell cycle checkpoint is induced by DNA damage. Cell cycle entry occurs after the DNA damages have been fully repaired, or alternatively, cells have two possible fates, to die or survive after a process of adaptation that allows cell division with unrepaired DNA lesions.

**Figure 2:**
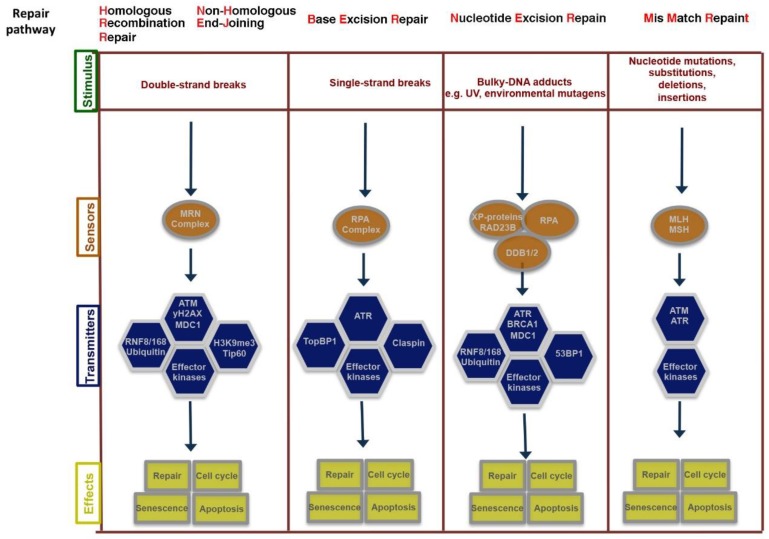
Schematic representation of the sensors, transducers and mediators involved in DNA damage response (DDR) pathways. DNA damage response is sensed and repaired by multi-protein complexes. Depending on the level of injury, the signaling triggered by the damage response will result in different cellular fates.

Genetic damage produced by either exogenous or endogenous mechanisms represents an ongoing threat to the cell. To preserve genome integrity, eukaryotic cells have evolved repair mechanisms specific for different types of DNA Damage (for an extensive review see [8,9]). However, regardless of the type of damage a sophisticated surveillance mechanism, called DNA damage checkpoint, detects and signals its presence to the DNA repair machinery. DNA damage checkpoint has been functionally conserved throughout eukaryotic evolution, with most of the relevant players in the checkpoint response highly conserved from yeast to human [10]. Checkpoints are induced to delay cell cycle progression and to allow cells to repair damaged DNA (Figure 1). Once the damaged DNA is repaired, the checkpoint machinery triggers signals that will resume cell cycle progression [11]. In cells, multiple pathways contribute to DNA repair, but independently of the specific pathway involved, three phase are traditionally identified: Sensing of damage, signal, and downstream effects (Figure 2). The sensor phase recognizes the damage and activates the signal transduction phase to select the appropriate repair pathway. For example, cells pose at least four independent mechanisms for repairing Double-Strand-Breaks (DSBs): Non-Homologous End-Joining (NHEJ), either classic-NHEJ or alternative-NHEJ, Homologous Recombination (HR), and single-strand annealing (SSA) [1,10,12,13]. Furthermore, highlighting the complexity of the DNA damage response, in mammals, at least four, in part, independent sensors can detect DSBs: Mre11-Rad50-Xrs2 (MRN), Poly ADP-Ribose polymerase (PARP), Ku70/Ku80 and Replication protein A (RPA) that binds single stranded DNA permitting the further processing of DSBs [1,14]. In the presence of DSBs, the activation of the DNA damage response and the mobilization of the repair proteins give rise to the formation of nuclear foci at the sites of damage. In yeast, the MRX-complex (Mre11-Rad50-Xrs2) is recruited at the site of DSBs [15]. Localization of MRX-complex to the damaged site is required to recruit and activate the protein kinase Tel1, which initiates DSBs signaling [13,16]. A similar mechanism is employed by MRN-complex in mammal cells (in which Nbs1 is the mammalian ortholog of Xrs2). MRN-complex orchestrates the cellular response to DBSs by physically interacting and activating the kinase Ataxia-Telangiectasia Mutated (ATM, the mammalian ortholog of Tel1). The signal is transduced by ATM that phosphorylates the histone variant Histone-2AX (H2AX) generating g-H2AX that promotes the recruitment of Mediator of DNA-Damage Checkpoin 1 (MDC1) protein at the site of damage. MDC1 amplifies the DNA-Damage Response (DDR) signal through the iterated recruitment of the MRN-ATM complex at the damage site that further phosphorylates adjacent H2AX molecules extending the γ-H2AX mark [13,16]. Additionally, MDC1 functions as an interaction platform for other DDR components including chromatin remodelers and ubiquitin ligase complexes [13,16]. The recruitment of these factors is essential to create a more open and accessible chromatin conformation to facilitate access at sites of DNA lesions and to allow ubiquitin-mediated accumulation of DNA repair factors, which will ultimately contribute to DNA repair pathways [13,16,17]. An integral part of the DNA damage response is the parallel induction of repair mechanisms and reversible cell cycle arrest that delays cell cycle progression to give cells time for DNA repair [11]. The Checkpoint kinases 1 and 2 (CHK1 and CHK2) are key downstream effectors of DDR signaling as they promote cell cycle arrest. ATM/ATR phosphorylate and activate the CHK1 and/or CHK2 kinase [18]. While CHK1 and CHK2 have overlapping substrate preferences, they contribute differentially to the maintenance of the cell cycle checkpoint. A central mechanism in the induction of the checkpoint-induced cell cycle arrest is the inhibition of cyclin-dependent kinase(s) (Cdk). In this mechanism, ATM and CHK2 are required to both stabilize and increase p53 DNA binding activity which in turn results in the induction of its several transcriptional targets, among which the Cdk-inhibitor protein p21waf1/cip [19,20]. A central target involved in the activation of the cell cycle checkpoint mediated by both CHK1 and CHK2 is the Cdc25 family of phosphatases (Cdc25A, B and C) [9]. Cdks are in an inactive state when phosphorylated at two inhibitory sites, Thr 14 and Tyr 15. Removal of these phosphates by Cdc25 phosphatases results in the activation of CDKs and cell-cycle progression [9]. Thus, CHK1/2-mediated phosphorylation of Cdc25 proteins results in their functional inactivation, preventing CDKs dephosphorylation and activation [9,21]. Overall, in mammal cells, CHK1 is thought to be the primary effector of the G2/M phase checkpoints, whereas CHK1 and CHK2 exert a cooperative role in the intra-S and G1/S checkpoints [22].

## 3. After Event Cleaning Job: RELEASE of the DNA Damage Checkpoint

The DNA Damage response elicits the activation of a highly complex and synchronized network of factors, such as kinases, phosphatases, transferases, and ligases [23,24,25,26,27]. Most of these enzymes add to remove functional groups that reversibly change the proteins fate or function [23,24,25,26,27]. Thus, when genome integrity is re-established the removal of these post-translational modifications is essential for a rapid checkpoint silencing and cell cycle progression [13]. Distinct DNA damage checkpoints at different stages of the cell cycle, such as G1/S, intra-S, and G2/M, have been described [28]. However, the exact dynamic and molecular basis of the recovery phase still remains not entirely clear. Recently, it has been shown that cell’s response to DSBs depends on its cell cycle phase and that checkpoint dynamics are phase-dependent [28]. In the G1 phase, DBSs completely halt the cell cycle only in the presence of high DNA damage levels. The most abrupt and complete halt to the cell cycle occurs during G2/M, and interestingly, cell cycle arrest is linearly correlated with the amount of DNA damage [28]. The S phase checkpoint is the more permissive to DNA damage and allows cell cycle progression, although at a greatly reduced rate [28]. However, multiple layers of complexity exist in order to prevent cell cycle progression in the presence of damaged DNA. Cell cycle progression occurs in a linear manner, in which each checkpoint functions as an additional layer of control of the previous checkpoint. Thus, the G1 checkpoint is important in cells that have been exposed to DNA damage in the G1-phase, as well as for those that have been adapted from the G2 checkpoint [29]. In this context, it is interesting to note that, conversely to the redundancy of factors and mechanisms that share a temporal and overlapping function in response to DNA damage, checkpoint recovery relies on the involvement of phase-specific factors [13]. The CDC25B is a S/G2 phosphatase that is thought to play an essential role in activating CDK1-cyclin B complexes at the entry into mitosis ([13] and references there in). CDC25B has been shown to cooperate with the polo-like kinase 1 (PLK1) in promoting the cell cycle resumption in G2 phase after DNA damage. In addition, recovery of the G2 DNA damage checkpoint appears to be distinct from G1. Indeed, both PLK1 and Cdc25B are not expressed in G1 and do not influence cell cycle resumption in G1 (Reference [13] and references therein). Essentially the same activation pathways promote mitotic entry in an unperturbed cell cycle and checkpoint recovery [30]. However, these pathways are thought to be differentially involved in these two processes. PLK1 is not essential for mitotic entry in cells progressing through normal cell cycles; it has been shown that the complete inhibition of PLK1 can only delay G2/M transition leaving the importance of PLK1 for mitotic entry during unperturbed cell cycle controversy [13,31]. Conversely, it is well established that initiation of the DNA damage response repress pro-mitotic machinery and leads to the inhibition of pro-mitotic kinases among which CDK1, Aurora A, and PLK1 [32,33,34]. Additionally, the degradation of Cdc25 and Bora, as well as of several other proteins involved in mitotic entry, is critical for cell cycle arrest [35,36]. While PLK1 is dispensable for the onset of mitosis in an unperturbed cell cycle, in sharp contrast PLK1, is essential for mitotic entry following recovery from DNA Damage-induced cell cycle arrest [37]. Cell cycle re-entry relies on the Aurora-A kinase and its co-factor Bora, which phosphorylates PLK1 at Thr210 in its activation loop; thus, Plk1 is activated and promotes mitotic entry by stimulating cyclin B1-Cdk1 activation [25,30,37,38]. PLK1 can promote cyclinB1/CDK1 activation by several mechanisms. Early works in *Xenopus* have established that Plx1 (PLK1) phosphorylates and activates Cdc25C, and this activates the Cyclin B–CDK1 complex. In vertebrates, the Cdc25 paralogues (Cdc25A, B and C), all have been shown to be target of PLK1 activity [39], but it remains poorly characterized, with Cdc25 phosphatase(s) the substrate of PLK1 during the G2 recovery. However, it has been suggested that G2 recovery is dependent on the specific isoform Cdc25B, which is stabilized after damage, while Cdc25A expression is reduced [37,40]. Beside its implication in the re-activation of cyclin-B1–CDK1 complex, PLK1 controls the silencing of DDR signals by inactivating the ATM/CHK2 pathway. Within the DNA damage response mechanism, 53BP1 is an adaptor protein required to tether several checkpoint components at the damaged sites, including CHK2 and ATM. In PLK1-mediated inactivation of the DNA damage checkpoint, it has been shown that PLK1 phosphorylated 53BP1 that thus fails to form foci after DNA damage [41]. Additionally, it has been shown that PLK1 also directly phosphorylates and inactivates CHK2 [41]. Thus, PLK1 negatively regulates the ATM-CHK2 branch of the DNA damage to inactivate checkpoint signaling and to control checkpoint duration [41]. Similarly, PLK1 negatively controls Claspin and CHK1 and the inactivation of these components results in a shutdown of the checkpoint [42,43,44]. Specifically, phosphorylation of Claspin by PLK1 creates a docking site for β-TrCP protein, resulting in the efficient ubiquitin-mediated degradation of this protein [42,43,44]. In conclusion, PLK1 is capable of driving entry into mitosis after DNA damage-induced cell cycle arrest and to promote checkpoint silencing and recovery.

## 4. DNA Damage and the Balance between Survival and Death 

A central question in cells responding to DNA damage is how DDR pathway controls cell fate decision. The accepted paradigm implies that the level of damage may trigger different responses; thus, low-level promotes the initiation of repair and the activation of survival mechanisms, whereas high-levels promote cell death. This concept includes the tacit assumption that, if the damage is irreparable, cells undergo apoptosis; however, there currently is not a clear biochemical mechanism for how cells distinguish between reparable and irreparable DNA damage. Evidence suggests that cells respond to DNA damage by simultaneously activating DNA repair and cell death pathways [45,46]; p53 protein and its functional ambiguity might play a central role in this context, given the ability of p53 to control the transcription of genes involved in either survival or death [47]. p53 influences several pathways, which are essential for progression through the cell cycle, including G_1_/S, G_2_/M and spindle assembly checkpoints [48]. Thus, it is not surprising that several signaling pathways can converge on p53 to control cellular outcomes. Among them, PLK1 was shown to physically bind to p53 inhibiting its transactivation activity, as well as its pro-apoptotic function [49]. As mentioned above, upon DNA damage, ATM/ATR alone lead to phosphorylation of several hundreds of proteins, among them p53 [50]. The Mouse Double Minute 2 protein (MDM2) represents one of the predominant and critical E3 ubiquitin ligase for p53, responsible for the dynamic regulation of p53 function [51,52,53,54]. MDM2 mediates p53 ubiquitination through a RING domain (Really Interesting New Gene domain). Additionally, p53 and MDM2 function in a negative feedback loop, in which MDM2 transcription is activated by p53 and under normal stress conditions, MDM2 maintains low levels of p53 protein [51,52,53,54]. Furthermore, it has been observed that MDM2 binds to the promoters of p53-responsive genes and form a complex with p53 by interacting with its transactivation domain, thus MDM2 mediates histone ubiquitylation and transcriptional repression of p53 targets genes [51,52,53,54]. Upon DNA damage, ATM/ATR either directly or through CHK1/CHK2 phosphorylate p53 (Reference [46] and references there in). Similarly, it has been shown that ATM phosphorylates MDM2 (References [46,55] and references therein); phosphorylation of p53 and MDM2 in response to DNA damage by ATM/CHK1/CHK2 is thought to abrogate the MDM2-p53 protein-protein interaction leading to p53 stabilization and activation. (References [46,55] and references therein). In this context, it is thought that a low-level of DNA damage causes a transiently expression and response of p53 whereas a higher-level of DNA damage leads to sustained p53 activation. Thus, upon DNA damage cell fate is determined by tunable threshold of p53. Previous studies have indicated that p53 may selectively contribute to the differential expression of pro-survival and pro-apoptotic genes, due to the higher affinity of p53 for its binding sites in promoter associated with cell cycle arrest, e.g p21/CDKN1A and lower affinity for those associated with apoptosis [47]. It has been shown that both pro-arrest and pro-apoptotic p53 target genes are expressed proportionally to the p53 expression levels [47]. It is conceivable that, upon DNA damage triggering apoptosis, cells must reach the pro-apoptotic threshold of p53 activity, whose level is determined by expression levels of p53 itself. Interestingly, it has been shown that lowering this pro-apoptotic threshold with inhibitors of antiapoptotic Bcl-2 family proteins sensitized cells to p53-induced apoptosis [47]. DNA damage can activate both p53 and Nuclear Factor kappa-light-chain-enhancer of activated B cells (NF-κB). A model describing the crosstalk between p53 and NF-κB was proposed by Puszynski and co-workers [56]. This work suggested that the diverse outcome of the p53/NF-κB crosstalk in balancing survival and death depended on the dynamic context of p53 and NF-κB pathways activation. It has been proposed that NF-κB activation preceding p53 activation render cells more resistant to DNA damage-related death [56]. Remarkably, data from gain and loss of function approaches demonstrated that sustained anti-apoptotic NF-κB activity in tumors might depend on mutant p53 activity [57]. Thus, the regulation of p53 and its downstream effects are likely to be dependent on its interaction with other signal transduction pathways, which may influence the final response to p53 activation. In addition to the above-discussed mechanisms that control p53′s duality in cell fate, site-specific phosphorylation of p53 also seems to be important in promoting its pro-apoptotic function. It has been observed that promoter selectivity of p53 is regulated by post-translational modifications [58]. In this context, the increased affinity of p53 to the regulatory regions of pro-apoptotic genes is related to its phosphorylation at serine-46 (ser46) [58]. Thus, in stress-conditions, phosphorylation of p53 at S-46 regulates its pro-death function through the induction of apoptotic genes such as *NOXA* [59] *PTEN* [60] and *TP53AIP1* [61]. Several kinases phosphorylate p53 on S-46 either directly (HIPK2, p38, PKCδ, and DYRK2) or indirectly through ATM/ATR, with the effect to promote upregulation of pro-apoptotic p53-target genes [62,63,64,65,66]. In addition to its role as regulator of the cell fate of genomically compromised cells, several studies have shown that p53 also directly impacts the activity of various DNA-repair pathways [67]. Thus, p53 appears a multitasking factor providing protection from cancer development by maintaining genome stability. In conclusion, p53 is a central component of the signaling network activated by the DNA damage response and the tight regulation and balance of its activity must be maintained to preserve the dynamic principle of the damage checkpoint.

## 5. Molecular Mechanisms of Checkpoint Adaptation

Cells have evolved a complex network to maintain the integrity of the genome. An essential event in the DNA damage response is represented by the cell cycle arrest that allows cells to repair damaged DNA before entering the subsequent phases of the cell cycle [11]. Thus, the expected consequence in the presence of DNA damage is that cell cycle re-entry will only occur following DNA repair [11]. However, cells can enter into cell cycle before repairing their DNA through a mechanism originally described as checkpoint adaptation [68,69,70]. While in mammal cells the molecular mechanism of checkpoint adaptation has remained controversial and largely unknown until recently, it has been extensively studied in *Xenopus* and yeast. Since the checkpoint adaptation and checkpoint recovery mechanism share keys factors, it is not surprising that components of the checkpoint adaptation response are highly conserved throughout the eukaryotic evolution [10]. In the yeast *S. cerevisiae*, analysis of deletion mutants indicates that multiple factors are involved in checkpoint adaptation, among them: Cdc5 (PLK1), Tel1 (ATM), and Mec1 (ATR) [16]. In response to different kinds of DNA damage, checkpoint activation promotes the recruitment of Tel1/Mec1 to the lesion site [15]. The Tel1/Mec1 kinases directly phosphorylate the adaptor proteins Rad9 and Mrc1 that are able to recruit and to activate the checkpoint Kinase Rad53, the structural homolog of human CHK2, but considered functionally similar to CHK1 [71]. Phosphorylation of Rad53 as well as that of CHK1 promotes cell cycle arrest [15,71,72,73]. Several observations indicate that inhibition of Rad53 plays a crucial role in the control of the adaptation process; in particular, Rad53 over-activation was observed in diverse adaptation-defective mutants [73]. Moreover, it has been shown that Cdc5-mediated phosphorylation of Rad53 is required for checkpoint adaptation [74]; consistently with the finding that a dominant negative Rad53 mutant was shown to bypass the requirement of cdc5, in a cdc5 adaptation-defective mutant [73]. Finally, Rad53 de-phosphorylation mediated by both the phosphatases Ptc2 and Ptc3 has been shown to bypass the DNA damage checkpoint [65,72,75]. Thus, most of the common pathways involved in checkpoint adaptation inhibit Rad53 to promote entry into the cell cycle.

A consistent link between the Plx1 (PLK1) and Chk1 has been also observed in *Xenopus laevis* [76]. Persistent replication stress promotes the interaction between Claspin and Plx1, which causes the phosphorylation and release of Claspin from the chromatin and thereby Chk1 inactivation [76]. While checkpoint adaptation has been extensively studied in both lower and higher eukaryotes, its existence in mammal cells has long been considered controversial [10,77]. However, soon after the studies cited above, several authors reported a similar type of functional interaction between PLK1 and CHK1 in human cells. Overall these studies depict a model in which PLK1 phosphorylates and promotes SCF^β-TrCP^ ubiquitin ligase-mediated processing of Claspin, thereby promoting CHK1 de-phosphorylation and inactivation [43,44,78]. Based on these studies, *PLK1* has attracted a lot of interest for understanding the molecular mechanism controlling checkpoint adaptation. Thus, a number of experimental observations have provided mechanistic insight into the involvement of PLK1 in checkpoint adaptation. Interestingly, was observed that in the presence of DNA damage PLK1 degradation is required to achieve a proper G2 arrest [79], consistently with previous observations indicating that sustained PLK1 activity following DNA damage increases the fraction of mitotic cells [33]. In addition to Claspin, it was shown that in checkpoint adaptation WEE1 kinase is a direct downstream target of PLK1 (Reference [37] and references there in) WEE1 negatively regulates entry into mitosis by promoting the phosphorylation of CDK1, thus inhibiting the CDK1/cyclin B complex. PLK1 phosphorylates and leads to degradation WEE1, thereby promoting entry into mitosis [Reference 37 and references therein]. The requirement of PLK1 activity in cells entering in mitosis it has been elegantly confirmed by using a fluorescence-based probe for PLK1 activity at single cell level [80]. It has been reported that increased PLK1 activity is detected in cells entering mitosis in unperturbed cell cycle and when cells recover from DNA damage checkpoint by addition of caffeine that force a shutdown of the checkpoint [25,80,81]. An interesting observation arising from these studies is that, once PLK1 activity increases beyond a certain level, it overrides damage checkpoint regardless of whether DNA damage persists [80].

However, while a number of studies favor the notion of a central role of PLK1 to drive checkpoint adaptation, likely there are multiple factors that contribute to the DNA damage recovery. CDK1 is a key regulator of mitotic entry, and as discussed above, PLK1 itself can phosphorylate it. Thus, it is likely that signaling pathways able to influence Cyclin B/CDK1 activity in conjunction with PLK1 potentially might regulate adaptation [13,16,37].

## 6. Consequences of Checkpoint Adaptation

Cell cycle checkpoints and DNA repair mechanisms are important processes to maintain the integrity of the genome and the faithful transfer of genetic information to daughter cells [10]. This surveillance mechanism provides time to repair the damage, and only when repair has been successful, the checkpoint is extinguished and cells re-enter into the cell cycle [1,10,12,46,77,82,83]. In unicellular organisms, if DNA repair is not possible, cells can overcome DNA Damage through checkpoint adaptation [15,21,71,77,84]. Interestingly, mounting evidence indicates that this concept is not only found in unicellular eukaryotes like yeast but it might be extended also in multicellular organisms [10,16,76,77,85]. While the critical determinants of the outcomes of checkpoint adaptation are not yet precisely understood, checkpoint adaptation has several possible consequences. For instance most cells that undergo checkpoint adaptation die, whereas some cells survive; surviving cells face two different fates: Some cells will die in subsequent phases of the cell cycle, but a small number of cells will survive and divide with damaged DNA [References [85,86,87] and references there in]. In line with this model, it has been demonstrated that in repair-defective diploid yeast, nearly all cells undergo checkpoint adaptation, resulting in the generation of aneuploid cells with whole chromosome losses that have acquired resistance to the initial genotoxic challenge [84]. An important consequence of this finding was the demonstration that adaptation inhibition, either pharmacologically or genetically, drastically reduces the occurrence of resistant cells [87,88,89]. Thus, both in unicellular and multicellular organisms checkpoint adaptation might represent a mechanism that increases cells survival and increases the risk of propagation of damaged DNA to daughter cells [86,87,89]. Understanding this aspect is particularly important as a weakened checkpoint, it has been shown, enhances both spontaneous and carcinogen-mediated tumorigenesis [90,91]. Additionally, DNA damaging agents are widely used in oncology to treat many forms of cancer [92]. Unfortunately, resistance to these agents can result from a variety of factors that significantly reduce their efficacy in cancer therapy [93]. There is evidence that checkpoint adaptation may drive the selection of therapy-resistant cells (Reference [92] and references therein). A better understanding of the mechanisms that determine either survival or death following checkpoint adaptation might provide insight into the potential mechanisms for the failure of cancer therapies, thereby facilitating further improvement of current cancer treatments. 

## 7. Future Directions

Cancer is often regarded as an asexual evolution in which cancer cells arise through the sequential acquisition of beneficial mutations that should confer an increased fitness to the adapted cells [94,95,96]. Checkpoint adaptation serves as a mechanism by which cells become adapted to stressful conditions [16,77,84,85,89,92]. As described above, in this process the interaction between DNA repair pathways and cell cycle checkpoints determines cell fate decision and prevents neoplastic transformation. Preservation of integrity of multicellular organisms relies on these extra layers of developmental control. While the nature of what adaptation means for tumor cells in a multicellular organism remains puzzling, several observations indicate that the DNA Damage response may also affect the biology of the surrounding cellular microenvironment (for review see Reference [97]). In this process, the DNA damage response in cancer cells produces a paracrine signaling to induce changes in nearby microenvironment. However, DNA-damage response plays a crucial role, not only in cancers, but also in a wide variety of hereditary as well as non-genetic diseases [98,99,100,101,102]. A better understanding of how the DDR-driven signals are regulated and received by the surrounding microenvironment could represent an opportunity to understand how the systemic homeostasis controls cell fitness.
